# Autonomy in the Development of Stem Cell-Derived Embryoids: Sprouting Blastocyst-Like Cysts, and Ethical Implications

**DOI:** 10.3390/cells10061461

**Published:** 2021-06-10

**Authors:** Hans-Werner Denker

**Affiliations:** Universitätsklinikum, Institut für Anatomie, University Duisburg-Essen, Hufelandstr. 55, 45147 Essen, Germany; hans-werner.denker@uni-due.de

**Keywords:** stem cells, embryoids, blastoids, gastruloids, blastocyst, expanded potential stem cells, development, morphogenesis, self-organization, ethics

## Abstract

The experimental production of complex structures resembling mammalian embryos (e.g., blastoids, gastruloids) from pluripotent stem cells in vitro has become a booming research field. Since some of these embryoid models appear to reach a degree of complexity that may come close to viability, a broad discussion has set in with the aim to arrive at a consensus on the ethical implications with regard to acceptability of the use of this technology with human cells. The present text focuses on aspects of the gain of organismic wholeness of such stem cell-derived constructs, and of autonomy of self-organization, raised by recent reports on blastocyst-like cysts spontaneously budding in mouse stem cell cultures, and by previous reports on likewise spontaneous formation of gastrulating embryonic disc-like structures in primate models. Mechanisms of pattern (axis) formation in early embryogenesis are discussed in the context of self-organization of stem cell clusters. It is concluded that ethical aspects of development of organismic wholeness in the formation of embryoids need to receive more attention in the present discussions about new legal regulations in this field.

## 1. Introduction

**Figure 1:**
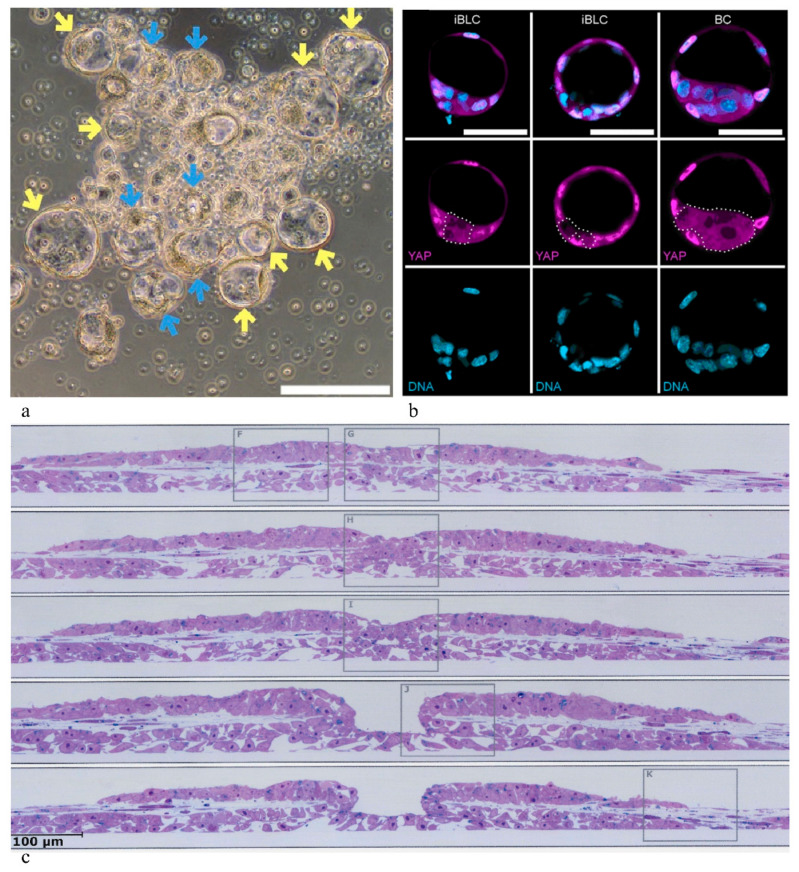
Embryoid structures autonomously developing in stem cell cultures. (**a**,**b**) Blastocyst-like cysts sprouting from a colony of mouse PPSCs: (**a**) morphology; (**b**) composing cell types of induced blastocyst-like cysts (iBLCs) in comparison with a blastocyst (BC). Embryoblast cells: magenta (YAP); DNA: light blue (from Kime et al. [71], with permission). (**c**) Embryonic disc-like colony formed spontaneously in rhesus monkey PPSC culture, showing a crater mimicking gastrulation-like cell ingression (serial sections) (from [73], with permission).

The experimental production of complex structures resembling mammalian embryos (or parts of them) from stem cells in vitro has recently become a rapidly growing research field (for reviews, see [1,2,3,4,5,6,7,8,9]). Depending on the degree of structural complexity, various types of such **embryoids** can be distinguished. For many years it has been known that structures which only vaguely resemble real mouse embryos in an early germ layer stage can develop from pluripotent stem cells (PPSC) in ascites or in suspension culture, i.e., the so-called **embryoid bodies** (for illustrations, see Figures 1 and 13 in [10]). During the last few years, however, numerous studies have been published on stem cell-derived constructs which resemble mouse and human embryos or embryo parts much more closely. Remarkably, this is emerging as a novel, booming research field, and various new terms have now been introduced to address these various entities (summarized and illustrated in [5]): **blastoids** (resembling blastocysts), **polarized embryo-like structures**, **gastrulating embryo-like structures**, **gastruloids**, **post-implantation amniotic sac embryoids**, and **asymmetric human epiblast**. A general term proposed for such human embryoids is **SHEEFs** (Synthetic Human Entities with Embryo-like Features) [11]. Rossant and Tam [6] have more recently proposed addressing such constructs with the general term “**stem cell-based embryo models**“, and to group them into two categories in order to emphasize aspects of potential viability (as possibly relevant for ethical implications arising when dealing with human stem cell-derived constructs), these two groups differing in their completeness, with or without extraembryonic cell types: (1) “**integrated stem cell-based embryo models**” would possess derivatives of the extraembryonic cell types (blastoids, blastoids with primitive endoderm, ETX/iETX embryoids), while (2) “**non-integrated stem cell-based embryo models**“ lack (part of) these extraembryonic cell types (gastruloids, amniotic sac structures, 2D micropatterned cultures).

As a result of the wealth of recent observations and publications, it is now widely realized that the morphogenetic potential of PPSCs should be of ethical concern when dealing with human cells, and it has become a topic to ask whether and where a line may be drawn between embryoids and real embryos, if any. The need for discussions on the ethical aspects of this peculiar stem cell potential for embryoid formation was indeed already seen many years ago, at the time when the first publications on human PPSCs appeared [12,13,14]; however, this aspect started to receive broader attention only much later [11,15]. The call for initiating a broad discussion on the ethical implications gained momentum when, in more recent years, methodologies were presented that allowed culture of real embryos (cleavage stages or blastocysts) up to post-implantation stages [16,17]. This was followed by studies which showed that when such improved methodologies were used for stem cell culturing, embryoids could be formed more regularly, and that some of these constructs could develop into complex structures which may come close to resembling, e.g., primitive streak or even more advanced stages (gastruloids), in the mouse model as well as in the human [18,19,20,21,22,23,24,25,26,27,28,29,30]. It was then asked whether these technical advances would be reason enough to question the validity of the limit so far set in many countries, for research with human embryos (or stem cell-derived embryo-like entities?) at the primitive streak/14-day stage [31,32]. Specifically with regard to stem cell-derived embryoids, a broad discussion has now been started by various institutions, with the aim to arrive at a consensus on whether new legislation should be initiated, and whether such new laws could be more liberal as to permit producing even more complete and advanced stage human embryoids for research [6,33,34,35,36], and which of the various embryoid models, and cultivation up to what stages, might be considered ethically permissible. This of course touches upon fundamental questions about the comparability of embryoids with real embryos and the dignity of embryos of various stages of development, about the respect they deserve, and whether there is reason enough to reconsider now the argumentation of the Warnock Report and perhaps even to change the derived regulations that are in force in the UK and in many other nations [31,32]. 

Any call for such changes in legislation should of course not be based only on the prospect of the new research possibilities but must consider all ethically relevant aspects connected with this new technology. In such considerations, an aspect often addressed is whether the degree of artificiality of embryoid constructs should be decisive; not the functionality (principal viability) of the “end product”, but rather how it came into existence, i.e., in a “natural” or an “artificial” way. Indeed, for the success of embryoid formation from stem cells, not only the type of cells of origin is important, but so are usually also details of the intricate manipulations that are applied in vitro. Such approaches are sometimes addressed as “embryo engineering”. Technical details that are found to be critical for permitting complex morphogenesis in these embryo engineering approaches are: specific culturing media supplements that regulate certain steps of lineage differentiation; various intricate manipulations, e.g., to use a defined small number of cells that are aggregated in separate wells to form initial clusters; to keep cell clusters non-attached or attached to specific kinds of substratum; to restrict the use of adhesive surfaces to certain stages of differentiation; or to construct a surrogate for axis pre-information for embryogenesis by combining different types of stem cell clusters in a specific way [18,22,24,25,26,27,37,38,39,40,41,42,43,44,45,46,47,48,49]; for reviews see [3,5,50,51], and for a discussion of the signaling and gene activation processes involved see [4,52,53].

Concerning ethical implications, such embryo-engineering approaches naturally focus our attention on the act of purposefully constructing these entities. Would this very act of “engineering” have any bearings on the dignity we have to ascribe to the emerging entities? Although this view is defended by some authors, it is refuted by others who argue that the functionality (possible viability) of the construct should be seen as the major point of concern [54,55]. When arguing in terms of Aristotelian philosophy, embryo-engineering approaches may remind us, with regard to aspects of potentiality, more of passive than of active potentiality of the entities in question. Passive potentiality is usually illustrated by the property owning to a block of marble such that this may be converted into a beautiful statue by a skilled artist. In contrast, any self-organizing capacity (morphogenetic potential) which would be exercised autonomously by this entity (without continuing informational input from the “artist”) would indicate active potential [56]. I will focus, in this article, on aspects of autonomy shown by embryoid structures developing from PPSCs in culture, which would indicate an acquisition of active potentiality. In order to put this into context, let us first briefly summarize some relevant facts about the morphogenesis of real mammalian embryos.

## 2. Some Relevant Facts about Mammalian Embryogenesis

Let us remember first what is known about the regular development of real mammalian embryos from the zygote. Mammalian development is, although of the regulatory (not a “mosaic”) mode and thus quite flexible, basically autonomous in the sense that it is not dependent on specific morphogenetic instructions from the outside, e.g., the uterus. A concept favored by many researchers for quite a while, i.e., that the uterus has to provide instructions for axis development at implantation, had to be abandoned in recent times due to experimental findings showing independence of the main morphogenetic steps from a uterine environment (reviewed in [55]; in addition to the references given there, a recent publication demonstrated development of mouse embryos in suspension culture up to limb bud stages [57]). Pre-information for the development of order along the future body axes appears to be provided by asymmetries of the oocyte/zygote, transmitted to cleavage and blastocyst stages by segregation, and modulated by cell–cell interactions subsequently. Morphological signs indicating any such asymmetries that may provide axial information are either missing or very discrete, in cleavage- or blastocyst-stage mammalian embryos (as discussed for the mouse by [58,59]; for some recent findings on segregation of Cops3 see below). It is still a matter of dispute whether the minute deviations from spherical or radial symmetry (which are indeed observed, such as the tilt/obliquity of the inner cell mass in the mouse blastocyst, and asymmetries in the trophoblast and primitive endoderm as seen in other species) do play a role in the determination of axes (discussed in [55]), and how they may originate. It is usually assumed that a process of “symmetry breaking” is crucial for an ordered development of germ layers and of a basic body plan with its body axes, at least in the mouse, but it has remained unclear where the signals for this symmetry breaking originate from and what their nature may be. Experimental findings make it increasingly clear that implantation in the uterus is not instrumental here, as was previously assumed by many authors (discussed in [55]). Asymmetry, including the formation of the anterior visceral endoderm (that plays a role in securing axis development) was, however, found to develop basically autonomously, not only in utero but also in vitro [60,61,62,63]. Recently, it was shown that early human embryos can develop into advanced stages in a simple gel matrix environment in vitro, independent of any specific morphogenetic instructions from the uterus [16,17]. In the mouse, even limb bud stages can be reached without uterine contribution, in suspension culture [57]. This clearly indicates that mammalian embryos are, in terms of systems analysis, complete developmental systems, possessing active (not just passive) developmental potential. They do, nevertheless, need a protective environment, since their ongoing developmental cascades can be disturbed by many noises (which would cause death of the embryo or malformations).

As far as the origin of information for the development of axes is concerned (first, the embryonic–abembryonic axis which is the precursor for the dorsoventral axis, later the anterior–posterior axis), existing experimental evidence is still limited and is being discussed controversially: (1) major role of pre-information provided by the oocyte/zygote cytoplasm, differentially segregated during cleavage to individual blastomeres (segregation theory), and (2) cell–cell interactions/signaling determining the path of differentiation exclusively (inside–outside theory, polarization theory) [58,59,64,65]. 

Recent research [66] seems to give reason for a renaissance for segregation theories (discussed in [67]). This work documents that the two first blastomeres of mouse embryos are not totally equipotent in most cases, contrary to previous assumptions, but that they rather differ in their epiblast-forming potential. Very interestingly, a molecular substrate for this imbalance between blastomeres was identified as the subcellular distribution of a gene product, the epiblast-related gene Cops3 [66]. Such findings must be seen in the context of theories on a developmental role of morphogens (or their precursor mRNAs) derived from the oocyte cytoplasm, which are asymmetrically localized there, so that these morphogens become segregated unequally to the blastomeres during cleavage. Theories which postulated segregation [68,69,70] had been largely abandoned, however, during the last 50 years, but now deserve to be reconsidered [67]. Whatever the relative importance of either segregation or cell–cell interactions, the resulting morula, as well as the blastocyst, is informationally a complete system and capable of exercising all morphogenetic steps autonomously without external information. This is nicely demonstrated by the cited recent observations on in vitro development using improved methodologies [16,17,57].

## 3. Autonomy in the Morphogenesis of Stem Cell-Derived Embryoids

If any oocyte/zygote-derived asymmetry is significant for early axis-formation processes in development, as the segregation theory postulates, such specific information must be missing in stem cell cultures; in contrast to early blastomeres, stem cells, even ES cells derived from early embryos, must be expected to have lost any oocyte-derived cytoplasmic asymmetry during passaging. In addition, it appears improbable that developmentally significant asymmetries of this type can be regained through reprogramming of fibroblasts, or at primed-to-naïve (and back) conversion of stem cells in culture as performed in some experimental approaches [71]. Nevertheless, remarkable self-organization processes have been and are being reported to occur in PPSC cultures, under certain conditions and even spontaneously, starting processes of this type independent of any specific embryo engineering maneuvers. Some examples are illustrated in Figure 1, blastocyst-like cysts recently described to sprout spontaneously in the mouse [71] (to be discussed more in detail further below). A particularly striking example are embryonic disc-like structures that have been observed already in 1996 to develop in dense cultures of marmoset monkey PPSCs (Figure 5 in [72]). Those authors described a primitive streak-like ingression center as well as amnion-like and yolk sac-like structures. Gastrulation-like ingression centers were also described to develop in rhesus monkey PPSC colonies (Figure 1c [73]). Anterior–posterior axis initiation has also been found to occur spontaneously in suspension cultures of mouse stem cell aggregates, a seminal observation that was received by many as a surprise [19] (gastruloid formation [20]). As has been discussed earlier [14], it seems to be significant for what is going on in cultures of stem cells that the asymmetry signals which probably govern morphogenetic patterning (the development of embryonic axes) in vivo can obviously be replaced by surrogate asymmetries in stem cell cultures, e.g., asymmetries in cell–cell and matrix densities that are always present in cultures and which arise stochastically [14].

Nevertheless, in many of the recent embryoid formation experiments using stem cells, it was found to be helpful to use a degree of “engineering” by providing some type of spatial information, e.g., to combine the various types of cells in an appropriate polar arrangement [23], or to apply physical constraints or specially engineered matrices [37,49,74]. Such experimental details are at least helpful for allowing morphogenesis to regularly reach stages largely resembling oocyte-derived embryos and their extraembryonic membranes [22]. They do not seem to be indispensable, however, as demonstrated by the observations on spontaneous/autonomous initiation of stem cell-derived embryoid morphogenesis just mentioned.

## 4. Spontaneous Budding of Blastocyst-Like Cysts in Stem Cell Cultures

A striking phenomenon of spontaneous morphogenesis, in clear contrast to specific embryo-engineering approaches, has indeed been described in the recent studies by Kime et al. [71,75], i.e., the formation of blastoids, so-called “**induced blastocyst-like cysts** (**iBLCs**)”. This development, remarkably, did not require specific physical manipulations but occurred spontaneously by a budding-like process, under their conditions (Figure 1a,b). Of note, considerable numbers of such iBLCs were produced in each culture vessel (see below). In their detailed analysis of the composing cell types, the authors emphasized the presence of three types of cells, autonomously taking typical positions in the iBLCs, i.e., trophoblast-like, primitive endoderm-like, and embryoblast-like cells, the latter showing properties of “**expanded**/**extended potential stem cells** (**EPSCs**)” or “**2C-like cells**” (discussed further below). In these experiments, Kime et al. [71,75] employed a methodology which they had previously developed and which allows conversion of primed state mouse PPSCs into the naïve state and v.v. with appropriate media supplements [76]. They started their cultures with a primed-to naïve state conversion (monitored using an X chromosome reactivation marker) of mouse epiblast stem cells. In such cultures, they observed the spontaneous formation of hemispherical cysts (**blastocyst-like hemispheres**) with certain features of blastocysts, i.e., a degree of blastocyst-like organization (although these half-cysts remained attached) and of lineage markers for trophoblast, embryoblast, and primitive endoderm cells.

In a second, more elaborated version of the culturing protocol, they applied a two-phase regimen, with a first phase including in the medium the SMAD2/3 signaling ALK5 inhibitor SB431542 (which inhibits primed state ActivinA/TGFß signaling); in a second phase, this small molecular inhibitor was omitted, but LIF and OMPT (a synthetic lysophosphatidic acid analogue which had previously been found to enhance embryogenesis in blastocysts by activating YAP [76]) were added. With this refined two-phase culturing system they now observed the formation of sprouting aggregates of a few (8–16) cells (called “iBLC precursors, iBLC-PCs”) which detached spontaneously and formed the free-floating blastocyst-like cysts (“**induced blastocyst-like cysts, iBLCs**”). The numbers of iBLCs budding per vessel were quite variable: at least 2–5 iBLCs per well of a 6-well plate, but often more than 30 (Table S1 in [71]). Remarkably, the formation of these iBLCs occurred **autonomously** in these cultures, just dependent on the media change, in a typical time course. It did not depend on any additional maneuvers in the sense of providing any pre-patterning, e.g., physical constraints or cell rearrangements (embryo engineering). During continuing cultivation, iBLCs self-organized to a remarkable extent and differentiated trophoblast as well as primitive endoderm-like cells (with the appropriate marker expression). Importantly, these iBLCs also possessed undifferentiated cells with stem cell-like (EPSC) characteristics.

Kime et al. compared the gene expressions of their sprouting iBLCs with those of normal blastocysts and found a number of differences in details of expression of various marker genes, as well as in morphology, in spite of the fact that main marker genes of embryoblast, trophoblast, and primitive endoderm were indeed expressed (for a commentary focusing on the molecular aspects see [77]). For example, they described a structural abnormality, i.e., GATA4-enriched cells bulging away. The blastocyst-like (BC-like) hemispheres mentioned in the initial part of the study were found to be more similar to blastocysts, with respect to marker expression, than the iBLCs. The authors concluded that pluripotent stem cells “can be reprogrammed to BC-like hemispheres with striking early embryonic implications, and … anticipate that cell conversions in such a context may be used to study early embryonic development in vitro”. This conclusion of course appears somewhat strained, at least as long as transcriptome analyses are missing. The iBLCs, although morphologically reminiscent of blastocysts and also showing many molecular similarities, appeared “imperfect and perhaps less neatly regulated than the BC-like hemispheres (e.g., PrE regulation, Xi reactivation, pluripotency)” [71].

### 4.1. Implantation Potential of Blastocyst-Like Cysts, and Endometrial Response

A remarkable observation in the work by Kime et al. [71] was that such iBLCs initiated early parts of an implantation cascade after transfer to a uterus in this mouse model, i.e., they elicited an implantation-like response (decidualization) in the endometrium and formed extraembryonic tissues. This was comparable to the initial phase of implantation events as seen with normal blastocysts transferred as a control. However, the extraembryonic membranes formed by iBLCs were grossly underdeveloped and disorganized, and the anlage of the embryo proper did not proceed on to differentiate a basic body plan. Instead, the whole artificial “conceptuses” became resorbed later on. In addition to blastocysts, other controls were similarly transferred to a uterus: mouse epiblast stem cell clusters, and embryoid bodies. These latter two controls did not implant nor elicit a decidual reaction, thus providing evidence that the iBLCs had shown a remarkable blastocyst-like behavior, i.e., a degree of specificity in their interaction with the receptive endometrium. This was obviously due to the fact that the iBLCs possessed trophoblast, in contrast to epiblast stem cell clusters and embryoid bodies. Overall, the observations by Kime et al. on implantation potential were quite comparable to results obtained with other stem cell-derived blastocyst-like constructs, as presented by Li et al. [39].

### 4.2. Extended/Expanded Potential Stem Cells, 2C-Like Cells

With regard to the developmental potentiality of the reported iBLCs, an important point appears to be that they possessed, in addition to trophoblast-like and primitive endoderm-like cells, a population of cells exhibiting molecular properties of so-called “extended/expanded potential stem cells” or “2C-like cells”, interesting sub-types of PPSCs that just recently started to receive attention in a number of labs [9,51,52,78,79,80,81,82,83,84,85,86,87,88]. In the following I will address all these PPSC subtypes together as **EPSCs** since they show many similarities in their developmental potentialities, although there are differences in their mode of derivation and their molecular characteristics. The presence of EPSCs in the cultures of Kime et al. [71] may indeed be important for the autonomous morphogenesis of these constructs.

Investigations on EPSCs gained momentum around 2015, with one of the first publications from the Torres-Padilla group on this peculiar subspecies of PPSCs [78]. These authors described a method of how “2C-like cells” (which had previously been found to occur spontaneously in stem cell cultures in small numbers [89]) can be induced to arise more frequently in vitro through down-regulation of the chromatin assembly activity of CAF-1. Ishiuchi et al. [78] reported that the EPSCs (“2C-like cells”) resembled blastomeres isolated from two-cell stage embryos, not only with regard to gene expression patterns but also to the capacity to reactivate transcription of endogenous retroviruses, as well as to the embryo-forming capacity gained during reprogramming by nuclear transfer to oocyte cytoplasm. Thus, it would appear reasonable to ask whether these EPSCs could express self-organization capabilities that exceed those known from traditional embryonic stem cell colonies, if tested in appropriate experimental settings. In the experiments by Kime et al. [71], the controlled conversion of the epiblast-type PPSCs into a naïve (EPSC) state and back appeared to be critical for initiating iBLC morphogenesis. The blastocyst-like constructs reported by Li et al. [39] were also derived from EPSCs. Another possibility would be that blastocyst-like structures may originate from oocyte-like cells, which may originate in such cultures and which can undergo parthenogenetic activation [90]. This latter possibility has not been investigated with regard to budding blastocyst-like structures in the recent studies.

In any case, EPSCs are much smaller than two-cell blastomeres, and like other PPSCs they cannot be expected to possess any axis pre-information provided via asymmetrical segregation of cytoplasmic determinants derived from an oocyte/zygote. As discussed before [14], self-organized early embryonic pattern formation can apparently be initiated in colonies of stem cells by surrogate signals, e.g., asymmetries in cell densities or physical constraints, as well as by the structure of the extracellular matrix. This is in agreement with computer modelings of pattern formation processes in development (the Turing/Meinhardt model) [14]. Such surrogate signals can be decisive for pattern formation in stem cell colonies in vitro (e.g., in the system used by Warmflash et al. [74] as discussed earlier [91]). For example, the type of extracellular matrix or of feeder layers was previously found to be critical for the formation of gastrulation-like craters (as shown in Figure 1c) in rhesus monkey PPSC colonies [92]. If the potential of EPSCs would be tested by, e.g., transferring the cells into an empty zona pellucida (providing a neutral environment excluding locally acting external asymmetry signals), it could be seen whether autonomous morphogenesis would be possible or impossible in this case [55]. Effects of the addition of an artificial local source of morphogen could be investigated. Such experiments could shed light on the question of what exactly the differences might be between a (totipotent) morula and a cluster of PPSCs/EPSCs formed under certain conditions. In order to determine how close the biological properties of EPSCs may come to those of blastomeres, it could appear interesting to study in various types of embryoids whether or not a regular primitive streak (and, thus, an incipient basic body plan) can be formed autonomously in vitro. It should be remembered that autonomous morphogenesis in marmoset monkey stem cell cultures was already reported, many years ago, to sometimes reach advanced stages, including primitive streak formation [72]. It would be of much interest to investigate the role of EPSCs in this type of morphogenesis in the marmoset monkey model [93]. Indeed, such experiments should not be performed in the human but with non-human primate PPSCs, in order to avoid any formation of a human basic body plan anlage in vitro [11,15,91].

## 5. Conclusions and Ethical Implications

What does all this tell us with regard to ethical implications arising when these embryoid formation technologies are applied with human stem cells? Undifferentiated PPSCs are not zygotes or embryos. However, as discussed, groups (colonies) of PPSCs can gain developmental autonomy in the process that is now usually addressed as self-organization, either on an organ level (organoids) or an early embryo level (blastoids, various higher organization levels of embryoids), depending on the epigenetic state of the initiating stem cells and on the local conditions provided in culture. For the start of these pattern formation processes and the subsequent morphogenesis, very simple asymmetries are obviously sufficient, as can occur in cultures stochastically [14]. The recent progress with formation of various types of embryoids from stem cells shows that these constructs are now reaching impressive complexity, with mouse as well as with human cells; that relatively well-structured embryonic as well as extraembryonic tissue (trophoblastic shell, amnion, yolk sac) anlagen are formed; and that a primitive streak stage can be reached in vitro. The anterior part of the primitive streak is known to act as an organizer, instrumental in the formation of a basic body plan (discussed in [14]). Organizer action marks individuation; this is the last stage of development at which the formation of identical twins is possible. It was for this reason that the primitive streak stage was set as a limit even in relatively liberal legal rulings for the use of human embryos for research, in many countries (as mentioned in the Introduction). The critical role of the primitive streak/organizer in individuation is the reason why now again a broad discussion has been initiated about the use of human embryos in research, and on the production and use of human embryoids, since the technological advances let it appear quite probable that human embryoids will soon be able to reach a complexity beyond the primitive streak stage that may approach viability. Primitive streak/organizer formation is not the only aspect of concern: embryoids (not only mouse but also human) are being proposed by some to be attractive models for the study of the biology of germ line cell formation [94], an aspect that is usually not covered by ongoing discussions on informed consent to be obtained from embryo and stem cell donors [35], but certainly has to be included.

If an in vitro system allows for the production of large numbers of stem cell-derived embryoids, as in the experiments described by Kime et al. [71], research along these lines may of course be facilitated. In my opinion, such experiments, in particular if involving EPSCs and leading to the formation of blastocyst-like or gastruloid constructs, should, however, not be extended to human stem cells, for ethical reasons, since what is initiated here in stem cell colonies are, on a large scale, self-organization processes which can be enabled to continue up to basic body plan (primitive streak etc.) stages, i.e., starting individuation. In a commentary, Pour and Nachman [77] recommended taking advantage of the methodology developed by Kime et al. [71] and to produce large numbers of human iBLCs from induced PPSCs (iPS cells) in infertility clinics, for diagnostic purposes, and possibly for the personalization of therapies, e.g., by studying the properties of iBLCs formed “from iPS cells derived from individuals with repeated failures of early pregnancy”. In my opinion, however, researchers and clinicians should rather be warned not to follow this path leading towards instrumentalization of early human embryonic constructs, since as a result of improvements in methodology, such types of embryoids are increasingly often showing developmental autonomy, and may approach viability (see literature overview in the Introduction) [11,15,91]. I thus urge us to critically reconsider the ethical implications of producing and of using human embryoid constructs that can gain organismic wholeness. Would there be alternatives for the use of human stem cells, avoiding the ethical dilemma but allowing basic research on embryoids to proceed? Obviously, in order to come as close as possible to the human system, non-human primate PPSCs may be used instead [93,95].

The main general message that developmental biology can contribute to the discussions concerning the ethics of research on human embryoid formation is that we need to understand better, and to consider more seriously, the biological basis of individuation. This includes questions on when and how a group of embryonic-type cells can gain morphogenetic independence, and can gain system properties of an organismic whole “in statu nascendi” [96], even though initially still showing little diversification of cells and tissues. Specifically, we need to pay attention to the question of under which conditions a group of stem cells may start the way to autonomy in the sense of gaining independence of pattern formation from outside signals, how this specific state of developmental autonomy can be detected, and how the process can be controlled. These questions open very important aspects to be urgently included in the ongoing discussions about new legal regulations concerning research on stem cell-derived embryoids, since we are talking here about the biological basis of individuation. Obviously, we will have to live with the prospect that the conclusions we may find necessary to be drawn here may force us to impose certain restrictions on our creator narcissism. The gaining of developmental autonomy should be considered a quantum leap with regard to the dignity to be ascribed to a colony of stem cells, moving it into the same ethical category as an embryo of that stage.

## 6. Note Added in Proof

After submission of this manuscript, some recent publications on stem cell-derived embryoid constructs have reported impressive progress of development towards even more advanced stages, in the mouse as well as in the human [97,98], thus giving additional support to the ethical considerations expressed in the present review. Also of note, the mentioned discussions about new legal regulations concerning the production and use of human embryoids, have resulted in the publication of new ISSCR Guidelines [99,100]. These Guidelines, however, do not address the aspects of developmental autonomy and organismic wholeness emphasized in the present review.
